# *Epidermal Growth Factor Receptor *and *K-RAS *status in two cohorts of squamous cell carcinomas

**DOI:** 10.1186/1471-2407-10-189

**Published:** 2010-05-11

**Authors:** Nancy Van Damme, Philippe Deron, Nadine Van Roy, Pieter Demetter, Alain Bols, Jo Van Dorpe, Filip Baert, Jean-Luc Van Laethem, Franki Speleman, Patrick Pauwels, Marc Peeters

**Affiliations:** 1 Department of Hepato-Gastroenterology, Digestive Oncology Unit, Ghent University Hospital, De Pintelaan 185 1K12IE, 9000 Ghent, Belgium; 2 Department of Head and Neck Surgery, Ghent University Hospital, De Pintelaan 185, 9000 Ghent, Belgium; 3 Centre for Medical Genetics, Ghent University Hospital, De Pintelaan 185, 9000 Ghent, Belgium; 4 Department of Pathology, Université libre de Bruxelles, Route de Lennik 808, 1070 Brussels, Belgium; 5 Department of Oncology, AZ Sint-Jan Brugge, Ruddershove 10, 8000 Bruges, Belgium; 6 Department of Pathology, Heilig Hart Ziekenhuis Roeselare, Wilgenstraat 2, 8800 Roeselare, Belgium; 7 Department of Gastroenterology, Heilig Hart Ziekenhuis Roeselare, Wilgenstraat 2, 8800 Roeselare, Belgium; 8 Department of Gastroenterology, Université libre de Bruxelles, Route de Lennik 808, 1070 Brussels, Belgium; 9 Department of Pathology, Ghent University Hospital, De Pintelaan 185, 9000 Ghent, Belgium; 10 MP is a Senior Clinical Investigator Research Foundation - Flanders

## Abstract

**Background:**

With the availability of effective anti-EGFR therapies for various solid malignancies, such as non-cell small lung cancer, colorectal cancer and squamous cell carcinoma of the head and neck, the knowledge of *EGFR *and *K-RAS *status becomes clinically important. The aim of this study was to analyse EGFR expression, *EGFR *gene copy number and *EGFR *and *K-RAS *mutations in two cohorts of squamous cell carcinomas, specifically anal canal and tonsil carcinomas.

**Methods:**

Formalin fixed, paraffin-embedded tissues from anal and tonsil carcinoma were used. EGFR protein expression and *EGFR *gene copy number were analysed by means of immunohistochemistry and fluorescence in situ hybridisation. The somatic status of the *EGFR *gene was investigated by PCR using primers specific for exons 18 through 21. For the *K-RAS *gene, PCR was performed using exon 2 specific primers.

**Results:**

EGFR immunoreactivity was present in 36/43 (83.7%) of anal canal and in 20/24 (83.3%) of tonsil squamous cell carcinomas. *EGFR *amplification was absent in anal canal tumours (0/23), but could be identified in 4 of 24 tonsil tumours.

From 38 anal canal specimens, 26 specimens were successfully analysed for exon 18, 30 for exon 19, 34 for exon 20 and 30 for exon 21. No *EGFR *mutations were found in the investigated samples. Thirty samples were sequenced for *K-RAS *exon 2 and no mutation was identified. From 24 tonsil specimens, 22 were successfully analysed for exon 18 and all 24 specimens for exon 19, 20 and 21. No *EGFR *mutations were found. Twenty-two samples were sequenced for *K-RAS *exon 2 and one mutation c.53C > A was identified.

**Conclusion:**

*EGFR *mutations were absent from squamous cell carcinoma of the anus and tonsils, but EGFR protein expression was detected in the majority of the cases. EGFR amplification was seen in tonsil but not in anal canal carcinomas. In our investigated panel, only one mutation in the *K-RAS *gene of a tonsil squamous cell carcinoma was identified. This indicates that *EGFR *and *K-RAS *mutation analysis is not useful as a screening test for sensitivity to anti-EGFR therapy in anal canal and tonsil squamous cell carcinoma.

## Background

With the recent progress in molecular biology, the tumorigenesis of cancer is becoming better understood, and clinical management has improved. Epidermal growth factor receptor (EGFR) has been validated as a therapeutic target in several human tumours, including colorectal cancer (CRC), non-small cell lung cancer (NSCLC) and squamous cell carcinoma of the head and neck (HNSCC). Ligand occupancy of EGFR activates the RAS/RAF/MAPK, STAT and PI3K/Akt signalling pathways, which together modulate cellular proliferation, adhesion, angiogenesis and migration [1,2].

Monoclonal antibodies directed against the extracellular domain of EGFR and small molecule inhibitors of the tyrosine kinase domain of the receptor have been evaluated in the treatment of several solid tumours including CRC, NSCLC and HNSCC [3]. Cetuximab, a chimeric humanized antibody, and panitumumab, a fully humanized monoclonal antibody have shown efficacy in combination with chemotherapy and also as monotherapeutic agents in CRC [4-6]. In NSCLC, approximately 85% of patients who responded favourably to gefitinib or erlotinib, two FDA-approved small-molecule EGFR-tyrosine-kinase inhibitors, were shown to have somatic mutations in the *EGFR *gene. Somatic *EGFR *mutations are primarily located in exons 18 through 21 around the ATP-binding pocket of the tyrosine kinase domain [7-10]. The most common mutations are short deletions in exon 19 affecting the amino acid sequence LREA (DelE746-A750) or a point mutation in exon 21 resulting in the amino acid change L858R. Increased *EGFR *gene copy number as determined by fluorescence in situ hybridisation (FISH) is known as a prognostic marker of progression-free survival and overall survival in HNSCC [11,12].

Several reports indicate that the presence of *K-RAS *mutations are a predictor of resistance to cetuximab and panitumumab therapy in metastatic colorectal cancer patients [13-16].

Cetuximab has been approved by the EMEA and FDA for HNSCC treatment. Recently, Vermorken *et al*. [17] described that cetuximab was effective in combination with platinum-based regimens for recurrent or metastatic squamous cell carcinoma of the head and neck.

Knowledge of the expression, amplification and mutation status of *EGFR *as well as downstream effectors such as *K-RAS *would help us to better understand the response of cancer patients to molecular targeted therapy.

Anal canal carcinoma is a relatively rare gastrointestinal malignancy with an increasing rate of incidence. The estimated number of new cases in the United States in 2009 will reach about 5290 patients (2100 males and 3190 females). It is estimated that (of the afore-mentioned number) 260 males and 450 females will die from anal canal carcinoma [18]. It is now apparent that the development of anal cancer is associated with infection by human papillomavirus (HPV), usually sexually transmitted [19,20]. In the literature, few data regarding *EGFR *and *K-RAS *status in squamous cell carcinoma of the anal canal is available [21-25].

HNSCC is the sixth most frequent cancer worldwide [26]. Despite current therapeutic modalities, many patients relapse or develop metastases, highlighting the need for new therapeutic targets. Several reports have described EGFR mutations in HNSCC patients, but these are heterogeneous, show ethnic differences in the frequency of occurrence, varying from 7% in Asians and to 0% to 4% in white patients [11,12,27-32]. In the literature, data regarding *K-RAS *status in HNSCC from the western world is scarce [33].

The aim of the present study was to analyse EGFR expression, *EGFR *gene copy number and *EGFR *and *K-RAS *mutational status in formalin-fixed, paraffin-embedded specimens from two cohorts of patients with squamous cell carcinoma, anal canal and tonsils.

## Methods

### Patient and sample characteristics

Formalin fixed, paraffin-embedded tissues from 51 squamous cell carcinomas of the anus and 24 squamous cell carcinoma of the tonsil were retrieved from the pathology departments of the participating institutions. The tissues were biopsied or resected between 1995 and 2006. Five micron thick sections were stained with haematoxylin and eosin for examination by light microscopy. The patient and sample characteristics are presented in Table 1. This study was approved by our local ethics committee. The number of evaluable cases for EGFR immunostaining, EGFR FISH, *EGFR *and *K-RAS *mutation analysis for the squamous cell carcinoma of the anus and tonsil are also presented in Table 1.

**Table 1:** Patient and sample characteristics.

	Anal Canal (n = 51)	Tonsils (n = 24)
Age (years)		
Median	60	58
Range	35-85	43-80
Gender (number)		
Male	25	18
Female	26	6
Specimens (number)		
Biopsies	15	2
Resection	36	22
Histological findings (number)		
Well differentiated SCC	17	5
Moderately differentiated SCC	20	13
Poorly differentiated SCC	14	6
Evaluable cases (number) for		
EGFR immunostaining	43	24
EGFR FISH	23	24
*EGFR *mutation analysis		
exon18	26	22
exon19	30	24
exon20	34	24
exon21	30	24
*K-RAS *mutation analysis	30	22

EGFR: Epidermal Growth Factor Receptor; FISH: Fluorescence In Situ Hybridisation; SCC: squamous cell carcinoma

### EGFR immunohistochemistry

EGFR immunostaining was performed using the Ventana system (Ventana Medical Systems Inc, Tucson, AZ). After deparaffinisation, five micron-thick sections were sequentially treated with inhibitor for 4 minutes and protease 1 for 6 minutes. Sections were then incubated with anti-EGFR mouse monoclonal IgG1 antibody (Clone 31G7, Zymed Laboratories, South San Francisco, CA, USA) for 32 minutes (1:100 dilution), after which they were incubated sequentially with amplifier A, amplifier B, biotinylated immunoglobulin, avidin-horseradish peroxidase and diaminobenzidine (DAB) for 8 minutes each. Sections were counterstained using haematoxylin for 6 minutes and bluing reagent for 2 minutes. All incubation steps were performed at 37°C.

Specimens were evaluated microscopically. Stains were considered positive when membrane staining of any intensity occurred in tumour cells. According to their staining intensity, positive samples were defined as weak (1+), moderate (2+) or strong (3+).

### EGFR-Fluorescence In Situ Hybridisation

Dual colour FISH was performed with the Vysis LSI EGFR Dual Color probe (Abbott Molecular Inc., Des Plaines, IL, USA) which hybridises to the band region 7p12 in SpectrumOrange and the centromere of chromosome 7 (7p11.1-q11.1, D7Z1 locus) in SpectrumGreen. FISH was carried out according to the protocol of the supplier.

For each slide, at least 20 neoplastic non-overlapping nuclei were scored for signals from both *CEP7 *and *EGFR *probes under the fluorescence microscope.

With a slight modification according to Cappuzzo *et al*. [34], patients were classified into five groups with ascending EGFR gene copy numbers. Briefly, disomy was defined as ≤ 2 copies in 90% of cells, trisomy as 3 copies in 10-40% of the cells, low polysomy as ≥ 4 copies in 10-40% of cells, high polysomy as ≥ 4 copies in ≥ 40% of cells, and *EGFR *amplification was considered to be present if > 10% of the nuclei contained multiple EGFR signals and the EGFR/CEP7 ratio was ≥ 2.

### *EGFR *and *K-RAS *mutation analysis

Genomic DNA was isolated from 3 × 50-μm formalin fixed, paraffin-embedded tissues specimens. These sections were cut and incubated with 500 μl of 1X phosphate-buffered saline (PBS) for 10 min at 80°C. After centrifugation (10 min - 14000 ×g), paraffin and PBS were removed. DNA was isolated using the Centra Puregene tissue kit according to the manufacturers instructions (Qiagen GmBH, Hilden, Germany).

The somatic status of the *EGFR *gene was investigated by PCR using primers specific for exons 18-21, encompassing the tyrosine kinase domain. For the *K-RAS *gene, PCR was performed using exon 2 specific primers. Subsequently, PCR fragments were analysed by direct sequencing in both sense and antisense direction. For ease of sequencing, M13 tails were attached to every primer pair. Primer sequences were as follows: *EGFR *exon 18 forward primer: CCTGAGGTGACCCTTGTCTCTGTGTTCTT, reverse primer: GAGGCCTGTGCCAGGGACCTTA, *EGFR *exon 19 forward primer: CGCACCATCTCACAATTGCCAGTTA and reverse primer: AAAGGTGGGCCTGAGGTTCA, *EGFR *exon 20 forward primer: cacactgacgtgcctctcc and reverse primer: tatctcccctccccgtatct, *EGFR *exon 21 forward primer: CCCTCACAGCAGGGTCTTCTCTGT and reverse primer: TCAGGAAAATGCTGGCTGACCTA, *K-RAS *exon 2 forward primer: cgtcctgcaccagtaatatgc and reverse primer: GTATTAACCTTATGTGTGACA. The following PCR program was applied: 5 min 95°C, 30 sec 95°C, 30 sec 62°C, 30 sec 68°C (with the last three steps repeated 42 times) and 7 min 68°C.

### Statistical analysis

Data were analysed using SPSS 15.0 software. Correlations between EGFR protein expression and gene amplification were evaluated using Pearson's χ^2 ^test. P-value < 0.05 was considered statistically significant.

## Results

### EGFR expression in squamous cell carcinomas of the anal canal and tonsils

**Figure 1:**
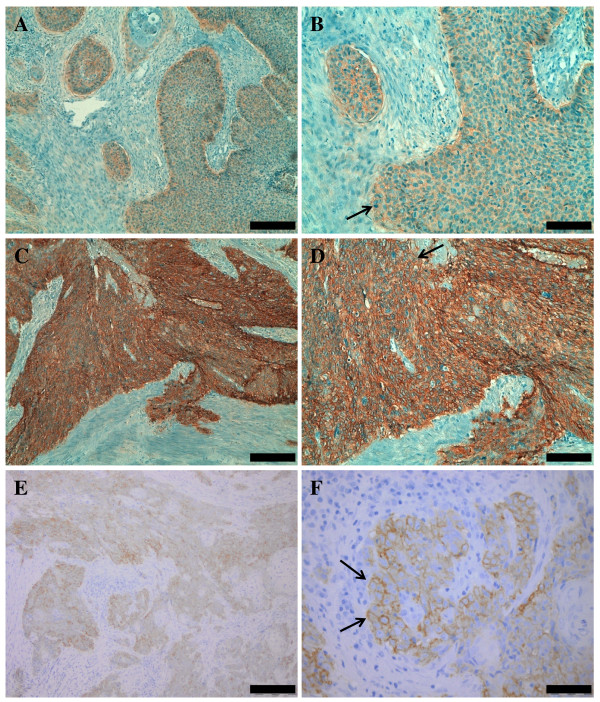
**EGFR immunostaining**. Anal canal squamous cell carcinoma, A to D. A. Weak immunostaining magnification x 100; B. Weak immunostaining magnification x 200; C. Strong immunostaining magnification x 100; D. Strong immunostaining magnification x  200. Tonsil squamous cell carcinoma, E and F. E. Immunostaining magnification x 100; F. Immunostaining magnification x 400. Arrows indicate membrane staining. For A, C and E, bar indicates 200 µm; B and D, 100 µm; and F 50 µm

The immunohistochemical findings of EGFR expression are summarized in Table 2. EGFR expression analysis could be performed on 47 of 51 anal canal specimens, due to lack of sufficient material from the remaining four specimens. Immunoreactivity to EGFR was not interpretable in 4 of the 47 cases. From the remaining 43 cases, 36 showed immunoreactivity to EGFR (83.7%). Among them, 29 (67.4%) cases exhibited moderate (2+) or strong (3+) staining intensities (Figure 1). There was no correlation between EGFR expression and patient age (P = 0.45) or differentiation grade (P = 0.82).

**Table 2:** Summary of Epidermal Growth Factor Receptor expression in anal canal and tonsil squamous cell carcinoma.

Immuno-reactivity	No. (%) of cases							
	**Anal Canal SCC (n = 43)**				**Tonsil SCC (n = 24)**			

	**-**	**1+**	**2+**	**3+**	**-**	**1+**	**2+**	**3+**

<5%	7 (16.3)	0	0	0	4 (16.7)	0	0	0
5-25%	0	4 (9.3)	2 (4.7)	1 (2.3)	0	6 (25)	0	1 (4.2)
26-50%	0	0	3 (7)	2 (4.7)	0	2 (8.3)	1 (4.2)	0
51-75%	0	1 (2.3)	4 (9.3)	2 (4.7)	0	2 (8.3)	1 (4.2)	2 (8.3)
>75%	0	2 (4.7)	3 (7)	12 (27.9)	0	0	2 (8.3)	3 (12.5)
Total	7 (16.3)	7 (16.3)	12 (28)	17 (39.6)	4 (16.7)	10 (41.6)	4 (16.7)	6 (25)

-: negative immunostaining 1+: weak immunostaining; 2+: moderate immunostaining; 3+: strong immunostaining; SCC: squamous cell carcinoma

EGFR expression analysis could be determined in all tonsil specimens. Twenty out of 24 showed immunoreactivity to EGFR (83.3%). Among them, 10 (50%) cases exhibited moderate (2+) or strong (3+) staining intensities. There was no correlation between EGFR expression and patient age (P = 0.56) or differentiation grade (P = 0.52).

### EGFR gene copy number in squamous cell carcinoma of the anal canal and tonsils

FISH analysis was performed on the same samples that were used for immunohistochemical analysis. However, 8 anal canal specimens could not be investigated due to technical problems and in 20 samples insufficient signal intensity was obtained for proper interpretation. Of the remaining 23 specimens, none showed *EGFR *gene amplification. *EGFR *disomy, trisomy, low polysomy and high polysomy were detected in 4, 11, 6 and 2 anal canal tissues, respectively (Table 3). The FISH patterns did not correlate with EGFR immunostaining (P > 0.05).

**Table 3:** *EGFR *status in anal canal and tonsil squamous cell carcinoma.

*EGFR *gene copy numbers	Anal Canal SCC (n = 23) No. (%)	Tonsil SCC (n = 24) No. (%)
Disomy	4 (17.4)	4 (16.7)
Trisomy	11 (47.8)	13 (54.1)
Low polysomy	6 (26.1)	3 (12.5)
High polysomy	2 (8.7)	0
Amplification	0	4 (16.7)

SCC: squamous cell carcinoma

**Figure 2:**
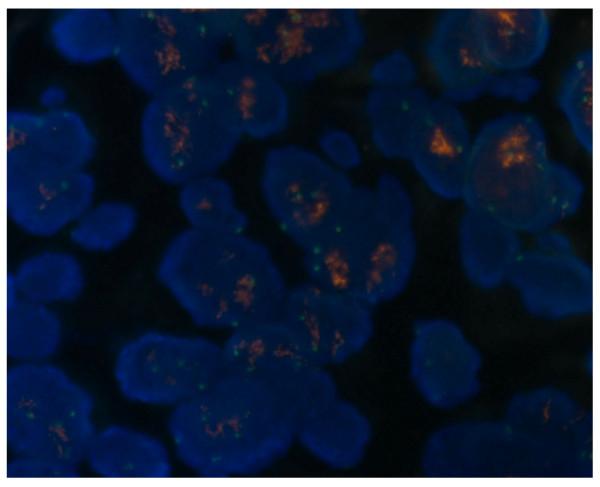
***EGFR *gene amplification by fluorescent in situ hybridization analysis in tonsil squamous cell carcinoma**. FISH analysis was performed using SpectrumOrange EGFR probe (red signal) with a SpectumGreen CEP7 probe (green signal).

All tonsil specimens could be investigated by FISH. Four specimens showed *EGFR *gene amplification (*EGFR/CEP7 *ratio ≥ 2) (Figure 2). In the remaining 20 tumours, disomy, trisomy and low polysomy were detected in 4, 13 and 3 tonsil tissues respectively (Table 3). Again, the FISH patterns did not correlate with EGFR immunostaining (P > 0.05).

### *EGFR *and *K-RAS *mutation analysis

In 10 of 51 anal canal specimens not enough material was available for DNA extraction. For three resection specimens sequence analysis failed for all investigated exons. Those same three specimens also failed FISH analysis indicating that most probably the fixation method of the available tissue did not allow for downstream DNA-based applications.

For *EGFR *mutation analysis exons 18 through 21 were examined. From the 38 anal canal specimens, 26 specimens were successfully analysed for exon 18, 30 for exon 19, 34 for exon 20 and 30 for exon 21. No *EGFR *mutations were found in the investigated samples. For *K-RAS *gene mutation analysis exon 2 was examined. Out of the 38 anal canal specimens, 30 were sequenced, and no mutation was identified.

**Figure 3:**
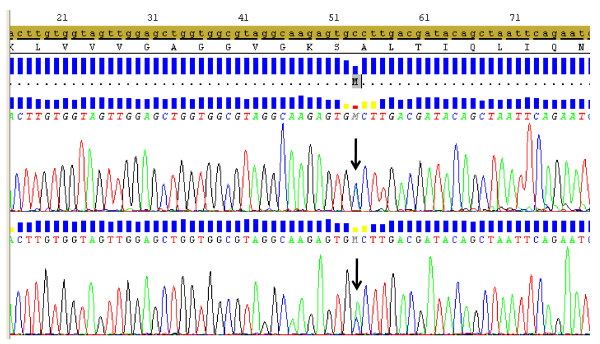
**Sequence chromatogram displaying the *K-RAS *mutation c.53C > A (p.A18D) in exon 2 (arrow) in a patient with tonsil squamous cell carcinoma, analysed by direct sequencing**.

All tonsil specimens were representative for DNA extraction. From those specimens, 22 were successfully analysed for exon 18 and all 24 specimens were successfully analysed for exon 19, 20 and 21. No *EGFR *mutations were found in the investigated samples. Out of the 24 specimens, 22 were sequenced for *K-RAS *exon 2 mutation and one mutation c.53C > A (p.A18D) was identified (Figure 3). This *K-RAS *mutated tonsil specimen showed no *EGFR *gene amplification and exhibited weak EGFR immunostaining.

## Discussion

With the availability of effective anti-EGFR therapies for various solid malignancies, such as NSCLC, CRC and HNSCC, the knowledge of *EGFR *and *K-RAS *status becomes clinically important.

Currently, few data regarding EGFR expression in squamous cell carcinoma of the anus are available [21-24]. Le *et al*. [21] found positive EGFR staining in all samples, Alvarez *et al*. [22] described EGFR immunoreactivity in 55% of studied tumours, Zampino *et al*. [23] reported positivity in 7 of the 12 evaluable cases and in the cohort of Walker *et al*. [24] 96% of the invasive anal canal cancers displayed EGFR immunoreactivity.

In the present study we showed immunohistochemical evidence of EGFR expression (i.e., at least 5% of tumour cells were positive) in 83.7% interpretable cases of squamous cell carcinoma of the anus. EGFR is overexpressed in most epithelial malignancies including HNSCC, ranging from 31 to 100% [32]. We showed EGFR expression in 20 out of 24 cases (83.3%) of squamous cell carcinoma of the tonsils. The results of the different immunohistochemical studies were however not consistent. These differences could be explained by the use of different antibodies, immunohistochemical techniques and scoring systems. Variations in EGFR immunoreactivity are also dependent on the fixation procedure and the storage time of unstained tissue sections [35].

In the search for which patients will benefit from anti-EGFR therapy, multiple studies investigating *EGFR *gene amplification have been performed. Increased *EGFR *gene copy number has been linked to poor prognosis in NSCLC [10] and HNSCC [11,12].

In our study, like Alvarez *et al*. [22] and Walker *et al*. [24], no *EGFR *gene amplification could be identified in anal canal squamous cell carcinoma samples. The prevalence of increased *EGFR *gene copy number in HNSCC varies in different studies, ranging between 13-58% [11,12,36]. In the present study, four tonsil squamous cell carcinomas showed *EGFR *gene amplification, defined as a ratio of *EGFR *gene copies to *CEP7 *gene copies of at least two in more than 10% of tumour cells. We found EGFR protein expression was independent of EGFR gene amplification. Although *EGFR *gene amplification was identified in only four cases of tonsil squamous cell carcinoma, it was not possible to correlate this finding with patient outcome.

Approximately 85% of NSCLC patients who responded favourably to gefitinib or erlotinib were shown to have somatic mutations in the *EGFR *gene [7-10]. About 90% of *EGFR *mutations affect small regions of the gene usually within exons 18 to 21, which encode for the EGFR tyrosine kinase domain. Anti-EGFR treatment can prevent activation of downstream signalling pathways such as the PI3K/Akt, RAS/Erk and STAT pathways, resulting in the inhibition of cellular proliferation and induction of apoptosis.

No prior study has investigated *EGFR *gene mutation status in squamous cell carcinoma of the anus. In our panel, exons 18 to 21, encoding the *EGFR *tyrosine kinase domain were investigated. No mutations in the *EGFR *gene were identified which excludes overexpression being the result of the presence of mutations.

Up to date there have been few studies searching for mutations in HNSCC and the results are contradictory. Lee et al. [27] found the mutation E746_A750del in 3 out of 41 Asian HNSCC patients (7.3%) and Na et al. [28] described several changes in 17 out of 108 Korean HNSCC patients (15.7%). Recently, one report analysed 91 Japanese HNSCC and 12 HNSCC cell lines for mutations in *EGFR*, *ErbB2 *and *K-RAS*. Only one silent mutation, C836T was found in exon 21 of *EGFR *in the UT-SCC-16A cell line. No other mutations were found [29].

Chung et al. [11] reported that no *EGFR*-activating mutations were found in 86 tumour samples from 82 American HNSCC patients. Temam et al. [12] also failed to detect any *EGFR *mutation in 134 French and American HNSCC patients. Lemos-Gonzalez et al. [30] analysed *EGFR *tyrosine kinase mutations from 31 Spanish HNSCC patients and none displayed a somatic *EGFR *mutation. Loeffler-Ragg et al. [31] screened 100 Caucasian HNSCC patients and only one displayed a novel, somatic *EGFR *missense mutation. From the same group Schwentner et al. [32] reported a rare *EGFR *mutation p. G796S in 2 out of 127 Austrian patients.

In our study, no *EGFR *mutations were found in tonsil squamous cell carcinoma, which confirms that *EGFR *kinase mutations are rare in Caucasian patients. It is known from studies in other tumour types (e.g. NSCLC) that somatic mutations in the tyrosine kinase domain of *EGFR *are much more common in adenocarcinomas than in squamous cell carcinoma [37]. Although the presence of activating mutations was first related to the ethnicity, it is now known that the frequency of *EGFR *mutations in NSCLC patients is not different in Western or Asian populations when the smoking habit is taken into account [9,38]. Although it is not clear that the pattern of *EGFR *mutations in NSCLC could be directly translated to HNSCC, the low frequency of *EGFR *mutations, and the fact that all but three patients included in our study are significant current smokers, could explain the absence of *EGFR *mutations in our subset of patients.

HPV-infection is a risk factor for head and neck, anal canal, cervical and vulvar squamous cell carcinomas. Recently, in head and neck and vulvar squamous cell carcinoma, *EGFR *mutations and protein overexpression were predominantly HPV-negative and associated with poorer prognoses [28,39]. Recently, Walker et al. [24] investigated EGFR expression in anal HPV-infected squamous intraepithelial lesions and/or invasive cancers. In both HIV-positive and HIV-negative patients the EGFR immunostaining increased from condyloma acuminata (HPV6 and 11 infected) through anal intraepithelial neoplasia 1, 2 and 3 till invasive squamous carcinoma (both infected with oncogenic HPV), highlighting the effects of oncogenic HPVs. Also, HIV-positive status contributes to augment EGFR expression levels involved in carcinogenesis. However, in our study the HPV-status and HIV-status was not systematically established. So, it would be of interest in future works to investigate both the HPV-status and HIV-status in anal squamous lesions.

Activating mutations in the *K-RAS *gene, which result in EGFR-independent activation of the mitogen-activated protein kinase pathway, are found in 35% of patients with CRC and in 15 to 30% of patients with NSCLC. The mutations are most frequently found in codon 12 and 13 of exon 2 of the *K-RAS *gene and are usually mutually exclusive with *EGFR *mutations [3]. Recently, several reports have indicated that *K-RAS *mutations are an important predictor of resistance to cetuximab [13-15] and panitumumab therapy [16] in metastatic colorectal cancer patients and are associated with an unfavourable prognosis.

Hiorns *et al*. [40] screened for activating mutations of the ras oncogene family in anal carcinoma using DNA amplified in vitro by PCR. Mutations were seen in two cases, both in *Ki-ras *codon 12. In our anal canal panel, exon 2 of *K-RAS *was investigated and no mutations were found. Mutations of the RAS family constitute one of the changes during cancer development. However, these mutations differ based on cancer type and ethnicity of the patients. In HNSCC patients from the western world these mutations were relatively infrequent [33] while in India they are very common [41]. In our tonsil carcinoma panel, one *K-RAS *mutation, c.53C > A (p.A18D) was identified. This specimen showed no *EGFR *gene amplification and had weak EGFR immunostaining. To look for the occurrence of this mutation, the COSMIC databank http://www.sanger.ac.uk/genetics/CGP/cosmic was screened and the c.53C > A mutation has been described in one Japanese lung adenocarcinoma patient [42].

## Conclusions

We can state that *EGFR *mutations were absent from squamous cell carcinoma of the anus and tonsils, but that EGFR protein expression was detected in the majority of the cases. *EGFR* amplification was seen in tonsil but not in anal canal carcinomas. In our investigated panel, only one mutation in the *K-RAS *gene of a tonsil squamous cell carcinoma was identified, indicating that *EGFR *and *K-RAS *mutations are infrequent in this cohort of squamous cell carcinomas. This indicates that *EGFR *and *K-RAS *mutation analysis is not useful as a screening test for sensitivity to anti-EGFR therapy in anal canal and tonsil squamous cell carcinoma.

## Competing interests

The authors declare that they have no competing interests.

## Authors' contributions

NVD participated in the design and coordination of the study, performed the statistical analysis and drafted the manuscript. PD provided clinical samples and helped to draft the manuscript. NVR participated in the design of the study, performed the mutation analysis and helped to draft the manuscript. PDM provided clinical samples and clinicopathological data and revised the manuscript. AB provided clinical samples and clinicopathological data. JVD provided clinical samples and clinicopathological data. FB provided clinical samples and clinicopathological data and revised the manuscript. JLVL provided clinical samples and clinicopathological data and revised the manuscript. FS performed the mutation analysis and revised the manuscript. PP participated in the design of the study, performed the immunohistochemistry and FISH analysis and helped to draft the manuscript. MP conceived of the study, participated in the design and revised the manuscript. All authors read and approved the manuscript.

## Pre-publication history

The pre-publication history for this paper can be accessed here:

http://www.biomedcentral.com/1471-2407/10/189/prepub
